# Genotype and Trait Specific Responses to Rapamycin Intake in *Drosophila melanogaster*

**DOI:** 10.3390/insects12050474

**Published:** 2021-05-20

**Authors:** Palle Duun Rohde, Asbjørn Bøcker, Caroline Amalie Bastholm Jensen, Anne Louise Bergstrøm, Morten Ib Juul Madsen, Sandra Læsø Christensen, Steffan Balling Villadsen, Torsten Nygaard Kristensen

**Affiliations:** 1 Department of Chemistry and Bioscience, Aalborg University, DK-9220 Aalborg, Denmark; asbjoernboecker@gmail.com (A.B.); caroline_bastholm@hotmail.com (C.A.B.J.); anlodx@gmail.com (A.L.B.); mijm94@gmail.com (M.I.J.M.); sandra-laesoe@hotmail.com (S.L.C.); steffan.b.v@hotmail.com (S.B.V.); tnk@bio.aau.dk (T.N.K.); 2 Department of Health Science and Technology, Aalborg University, DK-9220 Aalborg, Denmark; 3 Department of Agroecology, Aarhus University, DK-8830 Tjele, Denmark

**Keywords:** aging, fecundity, heat stress tolerance, *Drosophila* Genetic Reference Panel, rapamycin, genotype by environment interaction, side effects

## Abstract

**Simple Summary:**

Rapamycin is commonly used as an immunosuppressant, but also as an anti-aging medicine. Despite its widespread use, results suggest that there is large variability in drug efficiency among patients, and limited knowledge exists about potential side-effects. In the present study, we investigated the effects of rapamycin using the common fruit fly as model organism. Six genetically distinct lines were exposed to rapamycin, and the phenotypic consequence on fecundity, longevity and heat stress tolerance was quantified. Flies exposed to rapamycin had increased longevity and heat stress tolerance, however a side effect in the form of decreased fecundity was also observed. Our data clearly show that the costs and benefits of rapamycin treatment is strongly genotype dependent. These observations are important as they imply that a ‘one size fits all’ approach when it comes to rapamycin treatment is not advisable. Future studies should address the underlying genetic component that drive the drug response variability.

**Abstract:**

Rapamycin is a powerful inhibitor of the TOR (Target of Rapamycin) pathway, which is an evolutionarily conserved protein kinase, that plays a central role in plants and animals. Rapamycin is used globally as an immunosuppressant and as an anti-aging medicine. Despite widespread use, treatment efficiency varies considerably across patients, and little is known about potential side effects. Here we seek to investigate the effects of rapamycin by using *Drosophila melanogaster* as model system. Six isogenic *D. melanogaster* lines were assessed for their fecundity, male longevity and male heat stress tolerance with or without rapamycin treatment. The results showed increased longevity and heat stress tolerance for male flies treated with rapamycin. Conversely, the fecundity of rapamycin-exposed individuals was lower than for flies from the non-treated group, suggesting unwanted side effects of the drug in *D. melanogaster*. We found strong evidence for genotype-by-treatment interactions suggesting that a ‘one size fits all’ approach when it comes to treatment with rapamycin is not recommendable. The beneficial responses to rapamycin exposure for stress tolerance and longevity are in agreement with previous findings, however, the unexpected effects on reproduction are worrying and need further investigation and question common believes that rapamycin constitutes a harmless drug.

## 1. Introduction

Development of new pharmaceuticals aimed to help humans suffering from conditions requiring medical intervention is heavily commercialised. It is a multi-billion dollar industry that has many stakeholders and large interests are at risk when new drugs are developed, tested and made available on the market. The path from developing a new drug, to obtain the permission to produce and finally to commercialise it, is long and strongly controlled. However, sometimes, unwanted side effects, adverse interactions with other drugs or genotype specific responses to a medical treatment can be hard to detect when testing new drugs [1,2], and such unexpected and unwanted effects can lead to recalling of already approved drugs [3].

Rapamycin and its analogue, everolimus, are approved for human use. They are macrolide compounds, which have immunosuppressive properties. In mammals, rapamycin acts as an allosteric inhibitor of TOR by binding to the protein FKBP12, which inhibits the expression of TOR complex 1 [4]. The downstream processes of TOR complex 1 include the regulation of the initiation of protein synthesis, biogenesis of ribosomes, synthesis of nucleic acids and autophagy [4,5]. Rapamycin has been proven effective against lung cancer [4] and it is also effectively used for coating of coronary stents, prevention of organ transplant rejection, and treatment of other lung diseases such as lymphangioleiomyomatosis [6,7,8]. Further, numerous studies with model species, including yeast [9], *Drosophila* [10,11,12] and mice [13,14,15], have revealed increased lifespan associated with rapamycin intake. Aging is a major risk factor for a wide range of diseases, including cardiovascular diseases, cancers, and neurodegenerative diseases, thus pharmacological compounds that counteracts the effects of aging are of novel importance [16]. Rapamycin constitutes an example of a drug that has been suggested to counteract many of the symptoms associated with age-related illnesses, such as Alzheimer’s disease, and reduced cognitive abilities [4,17].

Individuals are genetically different and have experienced different environments throughout life. This may severely impact on the responses to medical treatment and it has been suggested, that 20–90% of the variation seen in the response to a medicine in humans is attributable to genetic differences among patients [18]. Such knowledge is a driver of the rapidly developing field of precision medicine [19]. However, in the process of testing a new drug it is standard practice to investigate its efficiency and potential unwanted side effects on one or few genetic backgrounds of cell cultures or laboratory species. This provides limited possibility to investigate the impact of genetic background on drug efficiency and genotype specific unwanted side effects. Therefore, small model organism like *Drosophila melanogaster*, have been suggested as promising model system for testing pharmacological interventions [20,21,22,23].

Here we tested the effect of rapamycin on six genetically different *D. melanogaster* lines from the *Drosophila* Genetic Reference Panel (DGRP) [24,25]. The DGRP was established from a wild caught out-crossed population from Raleigh, North Carolina, U.S.A. It consists of 205 inbred lines each created though 20 generations of consecutive full sib mating. This procedure produces isogenic lines where individuals within a line are genetically identical (with an inbreeding coefficient of 1), which allows for testing numerus individuals with the same genetic background from each line. Further, the different lines are genetically diverged due to founder events and genetic drift. The DGRP has been assessed for numerus traits and differ markedly in behavioural, morphological and life-history traits (see Review by Mackay and Huang (2018) [26], and Anholt and Mackay (2018) [27]). Further, lines from the DGRP have been shown to respond markedly different to drugs such as methylamphetamine [28]. These characteristics of the DGRP system and the fact that all lines are full genome sequenced make it an advantageous system for investigating the genetic architecture of complex traits and genotype by environment interactions. Here we fed individuals from six randomly selected DGRP lines with rapamycin or a control treatment to investigate the impact of long-term rapamycin exposure (5–6 days or throughout life for the longevity experiment) on male longevity and male heat stress tolerance, and on female fecundity. Overall, we find strong evidence for line-specific consequences of rapamycin and unwanted side effects on fecundity. We also show that these finding are not a consequence of genetic differences in the TOR gene but likely attributable to small effects from a large number of genes; thus, response to rapamycin constitutes a complex quantitative genetic trait.

## 2. Materials and Methods

### 2.1. D. melanogaster Husbandry

From the original set of 205 inbred *D. melanogaster* lines from the *Drosophila* Genetic Reference Panel (DGRP) [24,25] a subset of six lines were randomly selected for the current study (RAL-32, RAL-348, RAL-630, RAL-748, RAL-801 and RAL-819). The DGRP lines were obtained from Bloomington *Drosophila* Stock Centre, and were kept at 23 °C under 12 h:12 h light-dark cycles in vials with 7 mL standard oatmeal–sugar–yeast–agar *D. melanogaster* medium [29] for two generations prior to the experiments. To produce experimental flies, fly density was partly controlled by allowing approximately 15 individuals per vial to reproduce for 24 h.

The individual flies used in this study were 24 ± 8 h old at the time of initiating experiments. Flies were sorted by sex under light CO_2_ anaesthesia within 24 h after emergence. Male flies were used for assessing longevity and heat stress tolerance whereas both males and females were used for the fecundity experiment (File S1).

### 2.2. Life History and Stress Resistance Assays

Two life history and one stress resistance traits were investigated (fecundity, longevity and heat stress tolerance), which are described in the sections below. Prior to each phenotypic assay the flies used were exposed to either a control treatment or the rapamycin treatment. Rapamycin is insoluble in water; therefore, ethanol was added to both treatments which consisted of 5% yeast extract, 5% sucrose, 50% demineralized water as well as 40% ethanol. For the rapamycin treatment 200 µM rapamycin (99.3% purity, CAS: 53123-88-9, Sigma Aldrich, Darmstadt, Germany) was added (this concentration was chosen based on previous findings [11]). For all experiments, the solutions were fed to adult flies using the capillary feeder (CAFÉ) assay [30]. In all experiments, the CAFÉ vials were kept within closed containers with water at the bottom to maintain a high humidity to reduce evaporation from the capillary tubes. The containers were kept in a climate chamber at 23 °C and 12 h:12 h light-dark cycle.

#### 2.2.1. Heat Stress Tolerance

We quantified heat stress tolerance by assessing the upper thermal limit [31] of male flies. For each DGRP line and treatment 60 males were distributed in six vials (ten per vial) containing 3 mL 3% agar medium. The flies were exposed to either the control treatment or the rapamycin treatment for five consecutive days using the CAFÉ assay. Hereafter, flies from each treatment and DGRP line were pooled and a total of 25 flies were randomly sampled per line and treatment. The flies were transferred to small empty glass vials (15 × 45 mm Screw Neck Vial, 4 mL). Each vial contained one individual and was tightly closed with a plastic screw cap. The vials were attached to a metal rack submerged in a circulating thermostatically controlled water bath (Lauda LCK 1892, LCK1892-14-0006, Lauda-Königshofen, Germany). The temperature of the water bath was increased with a rate of 0.1 °C/min from an initial temperature of 23 °C. Flies were continuously monitored and the temperature where no movement could be induced with a flashlight and a gentle knocking on the vials with a stick was noted as the upper thermal limit (CT_max_) [31].

#### 2.2.2. Fecundity

The effect of rapamycin treatment on female-fecundity was measured by distributing 60 males and 60 females into four vials (i.e., 30 flies per vial) containing 3 mL 3% agar medium for each DGRP line and treatment. The flies (both sexes) were exposed to either the control treatment or the rapamycin treatment for five consecutive days using the CAFÉ assay. Agar medium without nutrients was used because we wanted flies to obtain nutrients from the CAFÉ assay and not from standard nutritious *Drosophila* medium. Hereafter 25 pairs of one male and one female were transferred to individual vials containing 3 mL 3% agar medium with added black fruit colour to ease the quantification of eggs. Flies had access to either the control treatment or the rapamycin treatment using the CAFÉ assay. Every 24 h for three consecutive days the flies were transferred to new vials and capillary tubes were exchanged with fresh solutions. Fecundity was estimated as the total number of eggs laid per female across the three vials after three days.

#### 2.2.3. Longevity

Longevity was quantified as the number of days individual male flies lived. 200 males from each DGRP line and treatment were distributed equally in 20 vials containing 3 mL 3% agar medium, with access to either the control treatment or the rapamycin treatment using the CAFÉ assay. Agar medium without nutrients was used because we wanted flies to obtain nutrients from the CAFÉ assay and not from standard nutritious *Drosophila* medium. Once per day, dead flies in each vial were counted and removed. In this process capillary tubes were exchanged with fresh solutions. The flies were transferred to new vials with fresh agar once per week.

### 2.3. Statistical Analysis

All statistical analyses were performed in R (v. 4.2) [32]. For heat stress tolerance and fecundity we assessed the effect of rapamycin using a linear mixed model as implemented in the R package ‘lme4′ [33]. To approximate a Gaussian distribution the data were rank normalized. We fitted the model

y = g + t + g × t + e,(1)

where y was the rank normalized heat stress tolerance quantity or fecundity measurement, t was the fixed effect of treatment, g was a random DGRP line effect, g × t was a random genotype-by-treatment interaction effect, and e was the remaining residual. Statistical differences within DGRP genotypes between treatments was determined using Welch Two Sample *t*-test. The longevity data was analysed using a survival model implemented in the ‘survival’ package [34]. The data were visualised with Kaplan–Meier curves, and statistical difference in survival curves between treatment groups was assessed using the log-rank test.

## 3. Results

**Figure 1:**
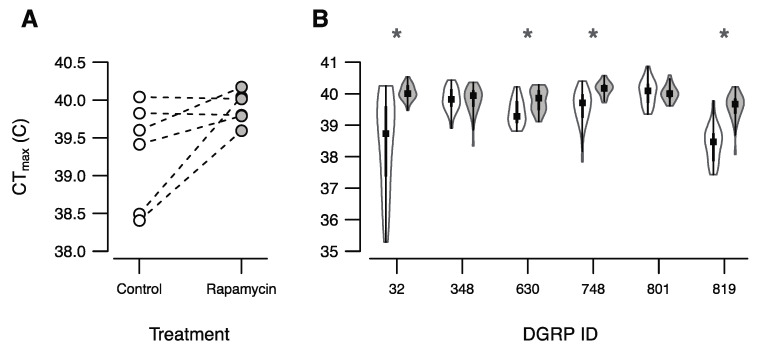
Effects of rapamycin treatment on male *D. melanogaster* CT_max_. (**A**) Interaction plot of DGRP genotypes across the two treatments. Points represent within line and treatment means. (**B**) Violin plots showing the distribution of heat knockdown temperature. Each violin represents the distribution of data with a boxplot inside where the median is indicated by a black square. Significant difference in heat knockdown temperature within lines are indicated with asterisks (*p*-value < 0.05).

The aim of the current study was to investigate the effect of rapamycin treatment on three fitness components: the upper thermal limit (CT_max_) in males, fecundity (number of eggs mated females produced) and male longevity. The DGRP-line average for CT_max_ of flies exposed to the control treatment ranged from 38.4–40.0 °C, whereas the range of values for flies exposed to rapamycin was 39.6–40.2 °C (Figure 1A). We found a strong and significant genotype-by-treatment interaction (*p*-value = $9.4\times 10^{-9}$), and a significant overall treatment effect (*p*-value = 0.031). Treatment with rapamycin significantly increased CT_max_ in four of the six DGRP lines investigated (Figure 1B), and interestingly, the range of CT_max_ values for the rapamycin treated flies were much narrower than the CT_max_ of the control flies.

**Figure 2:**
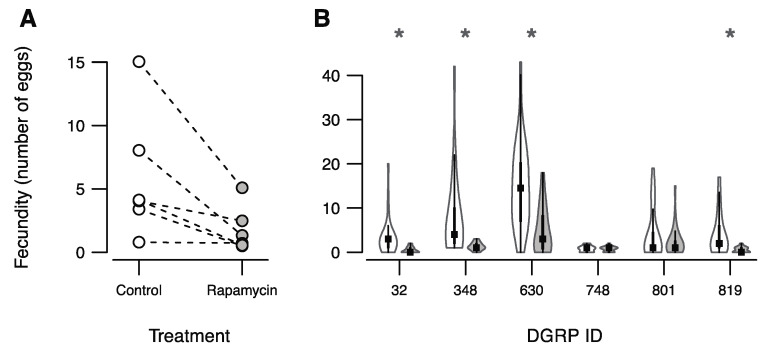
Effects of rapamycin treatment on *D. melanogaster* fecundity. (**A**) Interaction plot of DGRP genotypes across the two treatments. Points represent within line and treatment means. (**B**) Violin plots showing the distribution of fecundity as number of eggs. Each violin represents the distribution of data with a boxplot inside where the median is indicated by a black square. Significant difference in fecundity within lines are indicated with asterisks (*p*-value < 0.05).

Rapamycin treatment significantly lowered the number of eggs produced (Figure 2A, *p*-value = 0.007). Similar to the observation for heat stress tolerance, we also observed a strong genotype-by-treatment interaction effect for fecundity (*p*-value = 0.0003). The number of eggs produced by the control treated DGRP lines ranged from 15 eggs to an average of 0.8 eggs, and with rapamycin treatment this was lowered to 5.1–0.05 eggs (Figure 2A). Fecundity was significantly reduced in the rapamycin treated group for four of the six DGRP lines (Figure 2B).

**Figure 3:**
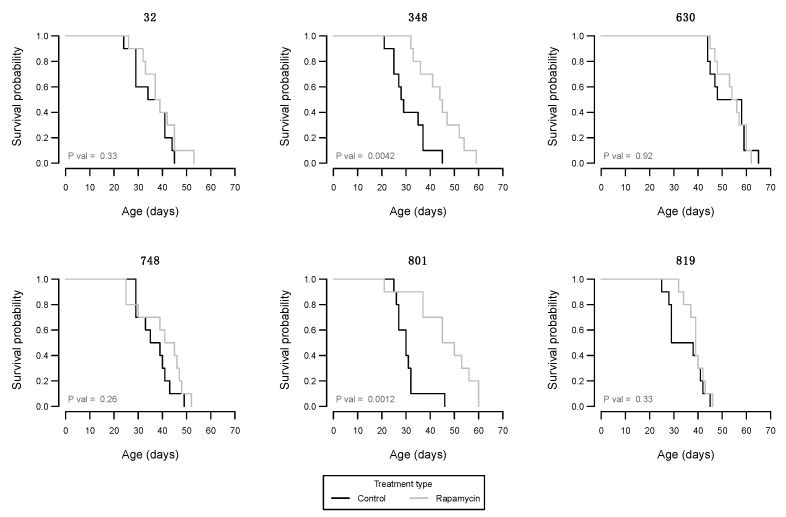
Kaplan–Meier plots of male *D. melanogaster* longevity within DGRP lines for flies exposed to the control treatment (black line) or the rapamycin treatment (grey line). *p*-values are from log-rank test.

The median lifespan of the control-treated male flies was between 28.5 days and 53.0 days, and with rapamycin treatment this range was between 38.0 days and 55.0 days. Thus, while the lower average lifespan was increased markedly by rapamycin treatment the upper lifespan was not. Only for two of the six DGRP lines investigated, we observed a significantly increased longevity with rapamycin treatment (Figure 3).

**Figure 4:**
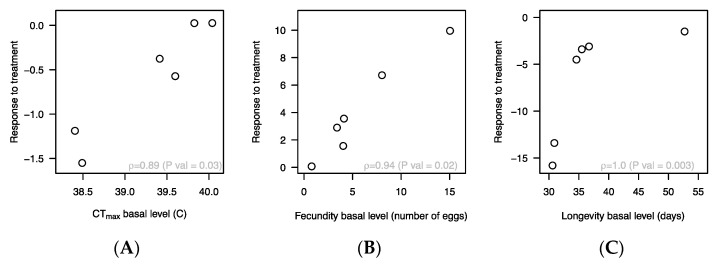
Trait-correlations between basal trait level (control treatment) and the phenotypic response to treatment (expressed as y_control_-y_rapamycin_, where y represent one of the three phenotypes) for CT_max_ (**A**), fecundity (**B**) and longevity (**C**). Results are based on the within treatment line means. Spearman’s rank correlation coefficients (ρ) with corresponding significance levels are shown for each trait. Numbers above each point are the DGRP line IDs.

**Figure 5:**
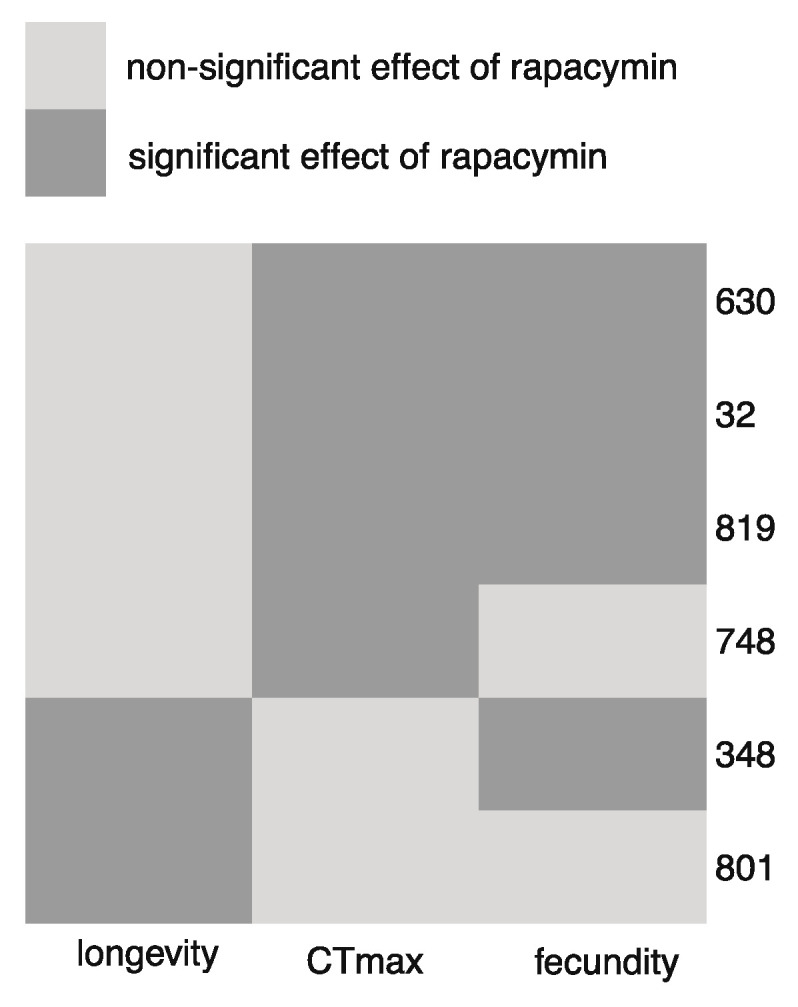
Result overview of significant within DGRP-line treatment effects (DGRP line ID is shown to the right on the figure).

The response to rapamycin-treatment (i.e., the phenotypic difference within line mean values between control treatment and rapamycin treatment) was strongly correlated with basal trait values (i.e., within line mean trait values) (Figure 4). The DGRP lines that were most heat tolerant (i.e., highest CT_max_) when exposed to the control treatment (basal level) responded little to rapamycin treatment for this trait whereas those lines that had the lowest basal CT_max_ responded strongly to rapamycin in the form of increased CT_max_ (Figure 4A). Likewise for longevity where lines that had low basal longevity responded most in the form of increased longevity when exposed to rapamycin (Figure 4C). For fecundity we observed that the lines with highest basal fecundity experienced the most pronounced decrease in fecundity when treated with rapamycin (Figure 4B). In combination, our results unambiguously illustrate that the phenotypic consequences of treating *D. melanogaster* with rapamycin is highly genotype specific (Figure 5) and further that the directional effect is trait specific.

## 4. Discussion

The main aim of this study was to investigate the effect of rapamycin on fecundity, heat tolerance, and longevity, and further if responses to rapamycin differed between lines that were genetically distinct. Using the DGRP lines for this purpose provide benefits because individuals from a given line are genome-wide homozygous meaning that a large number of genetically identical individuals can be tested across and within different treatments [24,25]. The DGRP system has been widely used to investigate the genetic basis of complex traits [26,27], and genotype by treatment interactions [28,35,36,37], however, this is to our knowledge the first study using the DGRP system to investigate rapamycin intake. In accordance with previous findings from *D. melanogaster* we see a reduction of fecundity estimated by the number of eggs produced over a 72 h time period in flies exposed to rapamycin (Figure 2) [11,12]. Furthermore, we found that the impacts of rapamycin on fecundity was highly line specific with four of the six lines having significantly reduced fecundity whereas no effect of rapamycin treatment was observed for two lines (Figure 2). We are not aware of studies on other species revealing these unwanted side effects of rapamycin on traits related to reproduction. In contrast, mTOR modulators including rapamycin has been suggested to ameliorate fertility issues in e.g., humans and mice [38,39]. For example, Dou et al. (2017) [39] showed that rapamycin treatment of mice can increase ovarian lifespan providing a potential tool to delay menopause. Moreover, when it comes to fertility-related diseases in humans including polycystic ovarian syndrome and endometriosis, rapamycin can have positive effects [38]. Thus, the finding that *D. melanogaster* produces less eggs when treated with rapamycin is somewhat contrary to findings in mammals illustrating the need for further studies on the impact of rapamycin on traits related to fertility and fecundity. In the interpretation of our fecundity data, it should however be kept in mind that our design does not allow for distinguishing between reduced fecundity and delayed fecundity. We assessed fecundity of females in a 72-h window early in life when flies were 5 to 8 days old. In this time-period the rapamycin treated flies had lower fecundity in four of the six lines. Since manipulation of TOR can affect developmental speed [40], some lines may have reduced fecundity early in life but increased fecundity at later ages. Longevity-fertility trade-offs is a common observation in studying life-history traits [11,41], thus lifelong fecundity should preferably be investigated in future studies on the effect of rapamycin on fecundity and longevity.

The heat stress tolerance assessed in a temperature-ramping assay in our study revealed that CT_max_ was significantly higher in male flies that had been exposed to rapamycin in four out of the six lines (Figure 1). Bjedov et al. (2010) [11] studied starvation resistance and found that both male and female *D. melanogaster* fed with rapamycin had an increased tolerance. These results suggest that rapamycin treatment in *D. melanogaster* increases robustness towards multiple environmental stresses. Chou et al. (2012) [42] found that the inhibition of mTOR results in a reduced production of heat shock proteins (Hsp). Based on the knowledge that Hsp are important for coping with high temperature stress [43,44] flies treated with rapamycin would then be expected to have reduced CT_max_ which is opposite to what we see. However, Duncan (2008) [45] found that while rapamycin blocks the translation of Hsp90, it does not affect the translation of Hsp70; thus not all Hsps are inhibited by rapamycin. Our study does not provide an explanation for why rapamycin causes increased heat tolerance in male flies but the involvement of TOR in several stress resistance pathways across a range of species is well known [46,47]. Interestingly, the positive impact of rapamycin on CT_max_ is not a universal finding across the investigated lines again showing strong impact of genotype on response to rapamycin in this trait. It is well known that the ability to cope with environmental stress including heat tolerance in *D. melanogaster* degreases with age [48,49,50,51]. Given that rapamycin treatment has anti-aging properties we suggest that the finding of increased heat tolerance in flies feed rapamycin is linked to a younger physiological age of these flies at the time of testing their CTmax (flies ca. 6 days old). This hypothesis needs experimental testing, and our data does not allow for investigating whether the finding of higher CT_max_ in rapamycin treated flies is linked to altered expression of Hsps in consequence of TOR inhibition.

Our longevity data confirmed previous findings from multiple species showing that rapamycin treatment has life-extending properties [46]. Bjedov et al. (2010) [11], Emran et al. (2014) [12], and Wang et al. (2016) [52] also found that rapamycin treatment of *D. melanogaster* with rapamycin significantly increased lifespan. Fok et al. (2014) [14] also found an 11% increase in the lifespan of male mice, using a corresponding concentration of 15.31 µM. Conversely, other *Drosophila* studies have demonstrated that treatment with rapamycin may shorten lifespan under certain drug dosages [53] or under nutritional stress [54]. In contrast to previous studies, we investigated impacts of rapamycin on several genetic backgrounds allowing for the investigation of genotype specific responses. As for the other traits assessed, longevity was also impacted in a highly line specific manner by rapamycin as only lines 348 and 801 had significantly increased longevity when treated with the drug (Figure 3). Interesting the median upper lifespan did not differ markedly between control and rapamycin treated flies (53.0 and 55.0 days, respectively) while the median lower lifespan was much higher for the flies exposed to rapamycin (28.5 and 38.0 days, respectively). This suggests that the upper lifespan limit is more constrained than the lower limit, which have also been suggested to be the case in humans [55].

We cannot rule out the lifespan of the flies and the phenotypic values for other traits investigated have been affected by the high ethanol concentration in the feed. Chandler et al. (2018) [56] investigated the effect of consumed ethanol on the lifespan of *D. melanogaster* with a maximum concentration of 15% and discovered a 15% reduction in lifespan. However, average male lifespan of the six DPRP lines investigated here does not deviate from estimates in the literature [57,58] and ethanol concentrations in natural *Drosophila* habitats (decomposing fruit) can be high [59]. This combined with the fact that both our experimental treatments had the same ethanol concentration suggest that this is not a big issue for interpretation of results from the current study.

Our experiment showed that treatment with 200 µM rapamycin significantly increased longevity and heat stress tolerance in male *D. melanogaster*. In contrast, fecundity was reduced in the rapamycin treated flies. For all traits we observed strong genotype by treatment interactions. Interestingly, we also found that for heat tolerance and longevity lines with high basal trait values for these traits responded little to rapamycin treatment whereas poor performing lines benefit more from rapamycin treatment (Figure 4A,C). We also observed that lines with high basal fecundity experienced the strongest cost in terms of reduced fecundity when treated with rapamycin (Figure 4B). Similar findings showing plastic responses to environmental changes being associated with basal trait levels have been reported for responses to thermal acclimation [60]. These results suggest an upper limit to performance that is hard to break by rapamycin and therefore the potential of rapamycin treatment seem larger for individuals with low fitness.

Our results have several implications: (1) when assessing effects of a medical drug multiple genetic backgrounds should be investigated because effects are typically genotype-specific, (2) several traits should be investigated because the effects of the medication is trait specific, (3) effects of rapamycin are not only genotype specific, but the genotype specificity is trait-specific (Figure 5) and (4) trait values of a given line (genotype) to a large extent predict costs and benefits of rapamycin exposure suggesting e.g., that positive effects of rapamycin is likely to be observed mainly in individuals with low fitness. Altogether these findings add to the complexity of medication exposure in general and rapamycin treatment specifically and warrant against conclusions based on testing one genetic background and one phenotype. Additionally, sex specific responses, which are not investigated in this study, should be further investigated. The strong genotype by treatment interactions observed here, and in another recent study on Ritalin treatment using the DGRP lines [28] also highlight the need and potential for tailoring treatment to the individual patient guided by the genetic profile.

The aim of this study was to investigate genotype specific responses to rapamycin and if unwanted side-effects of rapamycin was observed. Due to power limitations (with only six lines investigated) we were not able to perform a genome-wide association study investigating the genetic architecture of response to rapamycin treatment. However, we did look into whether the drug response variability observed in our study was driven by genetic differences within the gene encoding TOR. Using the genome-wide available genotypes [24,25] we extracted the TOR-gene, and by using Variant Effect Predictor [61] the protein effect was predicted. By doing that we observed a total of 102 genetic variants within TOR, whereof 20 were located in intron regions, 75 were synonymous variants, five variants were located in UTR ‘3 and two were predicted to be missense variants. Thus, only the two missense variants are of interest, as these causes changes in the amino acid sequence of the protein. However, those two variants were not segregating among the six randomly chosen DGRP lines, and therefore we cannot assess the important of genetic variation within TOR for rapamycin response. Hence, the observed line-specific responses to rapamycin is not due to genetic variation within the TOR gene, but is likely caused by small effect variants distributed across many loci, which is consistent with the perception that drug-response phenotypes constitute complex quantitative genetic traits (as demonstrated by several DGRP studies [28,35,62,63]. Future studies using the DGRP can be useful for pinpointing the genetic architecture of rapamycin response and reveal putative genes responsible for the reaction to rapamycin treatment. We also advocate that future studies investigating rapamycin in DGRP lines obtain baseline levels of TOR activity as well as after exposure to rapamycin, in particular as previous study has demonstrated that flies exposed to rapamycin display reduced TOR activity [11]. This would allow for deeper insight into the mechanistic basis of our findings.

## Data Availability

Available in supplementary material.

## Supplementary Materials

The following are available online at https://www.mdpi.com/article/10.3390/insects12050474/s1, File S1: Data.
